# 凝血与纤溶标志物在肺癌血栓栓塞患者中的临床应用价值研究

**DOI:** 10.3779/j.issn.1009-3419.2018.08.03

**Published:** 2018-08-20

**Authors:** 阳 付, 玉梅 刘, 亚雄 金, 虹 江

**Affiliations:** 610041 成都，四川大学华西医院实验医学科 Department of Laboratory Medicine, West China Hospital, Sichuan University, Chengdu 610041, China

**Keywords:** 血栓, 肺肿瘤, 凝血标志物, 纤溶标志物, 诊断效能, Thrombosis, Lung neoplasms, Coagulation biomarker, Fibrinolysis biomarker, Diagnosis power

## Abstract

**背景与目的:**

凝血与纤溶标志物能有效反映肺癌术后静脉置管患者体内止凝血各系统的功能紊乱，其水平变化与患者高凝状态密切相关。本研究旨在分析凝血与纤溶标志物在肺癌术后静脉置管患者中的变化趋势，评价其在肺癌血栓性疾病中的诊断效能，探讨其在肺癌深静脉置管患者凝血与纤溶平衡改变中的临床意义。

**方法:**

收集肺癌术后静脉置管患者118例，其中肺癌血栓栓塞患者29例、对照组89例。分别检测血浆中血栓调节蛋白（thrombomodulin, TM）、凝血酶-抗凝血酶Ⅲ复合物（thrombin-antithrombin complex, TAT）、纤溶酶-α2纤溶酶抑制剂复合物（α2-plasmin inhibitor-plasmin complexes, PIC）、组织型纤溶酶原激活剂-抑制剂1复合物（tissue plasminogen activator-inhibitor complexes, t-PAIC）和凝血酶原时间（prothrombin time, PT）、活化部分凝血活酶时间（activatedpartial thrombo plastin time, APTT）、凝血酶时间（thrombin time, TT）、纤维蛋白原（fibrinogen, FIB）、抗凝血酶Ⅲ（antithrombin Ⅲ, ATIII）、纤维蛋白原降解产物（fibrinogen degradation products, FDP）以及D二聚体（D-Dimer, D-D）的水平，分析其变化及血栓发生的诊断效能。

**结果:**

在肺癌术后静脉置管患者中，凝血与纤溶标志物TM、TAT、PIC、t-PAIC、D-D、FDP水平在血栓组高于非血栓组，差异均有统计学意义（*P* < 0.05）。诊断效能分析显示，TM、TAT、PIC、t-PAIC、D-D及FDP的曲线下面积分别为0.770、0.771、0.669、0.671、0.819和0.816，差异具有统计学意义（*P* < 0.05）。

**结论:**

肺癌术后置管患者体内凝血和纤溶活性增强，早期监测凝血和纤溶标志物可预防血栓的发生，减少肺癌患者术后血栓并发症的发生。

## Abstract (en)

**Background and objective:**

Coagulation and fibrinolysis biomarkers can effectively reflect the dysfunction of coagulation and anticoagulation system, and the changes of their levels were closely related to the hypercoagulable status. The aim of this study is to study the variation tendency of these coagulation and fibrinolysis markers and explore the diagnosis power and clinical value of these biomarker for thrombosis in postoperative lung cancer patients with deep vein catheterization.

**Methods:**

We selected 118 postoperative lung cancer patients with deep vein catheterization including 29 patients with thromboembolism and 89 patients in control group. Coagulation and fibrinolysis parameters [thrombomodulin (TM)/thrombin-antithrombin complex (TAT)/α2-plasmin inhibitor-plasmin complexes (PIC)/tissue plasminogen activator-inhibitor complexes (t-PAIC)] and traditional coagulation time[prothrombintime (PT)/activated partial thrombo plastin time(APTT)/thrombintime (TT)/fibrinogen (FIB)/antithrombin Ⅲ (ATIII)/fibrinogen degradation products (FDP)/D-Dimer (D-D)] were detected in both groups. We analyzed the variation tendency of these biomarkers and figured out the diagnosis powerfor thrombosis.

**Results:**

A statistically significant difference was available on the value of TM, TAT, PIC, t-PAIC, D-D, FDP between thrombosis group and non-thrombosis group (*P* < 0.05). TM, TAT, PIC, t-PAIC, D-D, FDP performed with an AUC of 0.770, 0.771, 0.669, 0.671, 0.819, 0.816, respectively (*P* < 0.05).

**Conclusion:**

An enhanced coagulation and fibrinolysis activity existed in lung cancer patients with deep vein catheterization after surgery, and early detection of coagulation and fibrinolytic biomarkers could prevent thrombosis and reduce postoperative thrombosis complications in patients with lung cancer.

肺癌是临床常见恶性肿瘤之一，目前治疗主要采取手术配合术后化疗、放疗的手段。但大多数化疗药物会引起静脉血管炎或迁移性的浅静脉炎^[1]^，药物外渗引起局部组织坏死，反复静脉穿刺给患者带来极大痛苦。故由深静脉穿刺置管技术来取代治疗中的反复静脉穿刺，可降低患者痛苦，但由静脉置管引起的血栓问题是值得我们关注的另一个重要问题^[2]^。

血栓调节蛋白（thrombomodulin, TM）、凝血酶-抗凝血酶Ⅲ复合物（thrombin-antithrombin complexes, TAT）、纤溶酶-α2纤溶酶抑制剂复合物（α2-plasmin inhibitor-plasmin complexes, PIC）、组织型纤溶酶原激活剂-抑制剂1复合物（tissue plasminogen activator-inhibitor complexes, t-PAIC）、D二聚体（D-Dimer, D-D）、纤维蛋白原溶解产物（fibrinogen degradation product, FDP）是血栓形成和纤溶系统激活的分子标志物，对肺癌患者体内凝血与纤溶的平衡改变有重要的临床指导作用^[3, 4]^。通过早期监测凝血标志物水平的变化来反映患者的血栓风险水平，针对性地进行预防抗凝措施，可降低肺癌患者术后血栓发生事件，改善患者预后。本研究旨在分析肺癌术后静脉置管患者的相关凝血/纤溶标志物血浆水平变化，评价凝血标志物在肺癌血栓性疾病中的诊断效能，探讨其对肺癌患者静脉血栓形成的临床意义。

## 1 材料和方法

### 1.1 一般资料

收集四川大学华西医院2016年10月-2017年10月肺癌术后深静脉穿刺置管患者118例，记录该患者的年龄、性别、血压、吸烟史、饮酒史、肺癌有无转移、糖尿病史以及甘油三酯（triglyceride, TG）、胆固醇（total cholesterol, TC）、高密度脂蛋白（high-density lipoprotein, HDL）、低密度脂蛋白（low-density lipoprotein, LDL）水平。根据中华医学会外科学分会血管外科学组发布的深静脉血栓形成的诊断与治疗指南分组：血栓性疾病组（*n*=29）和疾病对照组（*n*=89）^[5]^。

### 1.2 标本采集

在采用低分子肝素抗凝药物预防性治疗前，对纳入患者用枸橼酸盐抗凝管采血4 mL，3, 000 rmp离心10 min，收集乏血小板血浆，保存于-20 ℃冰箱待检。

### 1.3 仪器及试剂

凝血和纤溶标志物TM、TAT、PIC、t-PAIC检测试剂和检测仪器HISCL-5000购自日本希森美康（Sysmex）公司；传统凝血时间PT、APTT、TT、FIB、ATIII、D-D试剂购自德国西门子诊断试剂公司，检测仪器为希森美康（Sysmex）CS5100，FDP检测试剂购自希森美康（Sysmex）公司，检测仪器为希森美康（Sysmex）CS5100。

### 1.4 检测方法

TM、TAT、PIC、t-PAIC的检测原理为化学发光法；PT、APTT、TT、FIB、ATIII的检测原理为凝固法；FDP、D-D的检测原理是免疫比浊法。

### 1.5 统计学分析

应用统计学软件SPSS 16.0分析。用中位数以及四分位间距法表示凝血标志物结果，采用非参数分析检验比较组间结果。采用受试者工作特征曲线（receiver operating characteristic curve, ROC曲线）法确定灵敏度（sensitivity）、特异度（specificity）、阴性似然比（negative likelihood ratio, NLR）、阳性似然比（positive likelihood ratio, PLR）、阴性预测值（negative predictive value, NPV）、阳性预测值（positive predictive value, PPV）、最佳诊断点（最靠近左上角的ROC曲线的点，cut-off值），以及计算*Youden*指数。*P* < 0.05为差异具有统计学意义。

## 2 结果

### 2.1 临床基本资料

肺癌术后静脉置管患者发生血栓组与未发生血栓组两组间性别比例、年龄结构、血脂水平——TG、TC、HDL、LDL、吸烟史、饮酒史、糖尿病史、高血压史、肿瘤是否转移差异无统计学意义（*P* > 0.05），见表 1。

**1:** 肺癌术后插管患者血栓组与非血栓组临床基本资料比较 The main characteristics of patients in thrombosis group and non-thrombosis group with lung cancer

Index	Thrombosis group(*n*=29)	Non-thrombosis group(*n*=89)	*P*
Gender(Male/Female)	25/4	66/23	> 0.05
Age(yr)	61(53-64)	58(49-63)	> 0.05
TG(mmol/L)	1.36(0.98-2.11)	1.58(0.97-2.39)	> 0.05
TC(mmol/L)	2.68(1.94-3.59)	2.84(1.98-3.75)	> 0.05
HDL(mmol/L)	0.42(0.28-0.63)	0.46(0.25-0.77)	> 0.05
LDL(mmol/L)	1.16(0.74-1.86)	1.29(0.59-1.92)	> 0.05
Smoking(Y/N)	20/9	52/37	> 0.05
Drunk(Y/N)	13/16	33/56	> 0.05
Diabetes mellitus(Y/N)	3/26	8/81	> 0.05
Hypertesion(Y/N)	5/24	16/73	> 0.05
Metastasis	20/9	66/23	> 0.05
The data was described as median with interquartile range; TG: triglyceride; TC: total cholesterol; HDL: high-density lipoprotein; LDL: low-density lipoprotein.			

### 2.2 肺癌术后静脉置管患者血栓组与非血栓组常规止凝血检测指标PT、APTT、TT、FIB、ATIII、D-D、FDP水平比较

在肺癌术后插管患者中，发生凝血功能障碍的血栓组血浆D-D、FDP水平高于非血栓组，差异具有统计学意义（*P* < 0.05），见表 2。

**2:** 肺癌术后插管患者发生血栓组与非血栓组常规凝血时间比较 Comparison of traditional blood coagulation indicators in thrombosis group and non-thrombosis group with lung cancer

Index	Thrombosis group (*n*=29)	Non-thrombosis group (*n*=89)	*P*
PT (s)	11.7 (11.23-12.50)	11.65 (11.05-12.95)	0.823
APTT (s)	29.10 (26.53-32.15)	28.85 (25.38-31.33)	0.427
TT (s)	18.2 (17.7-19.1)	18.3 (17.5-19.1)	0.786
FIB (g/L）	3.99 (2.93-6.80)	3.29 (2.76-4.38)	0.116
ATIII (%)	73.6 (46.8-87.1)	85.9 (73.5-93.6)	0.073
D-D (mg/L)	3.24 (1.36-4.70)	0.84 (0.46-2.36)	0.000
FDP (mg/L)	7.5 (5.2-11.3)	2.5 (2.5-5.4)	0.000
The data was described as median with interquartile range.			

### 2.3 肺癌术后静脉置管患者血栓组与非血栓组凝血纤溶分子标志物TM、TAT、PIC、t-PAIC水平比较

在肺癌术后静脉置管患者中，血栓组凝血纤溶标志物TM/TAT、PIC、t-PAIC高于非血栓组，差异均有统计学意义（*P* < 0.05），见表 3。

**3:** 肺癌术后插管患者发生血栓与非血栓组凝血与纤溶标志物的比较 Comparison of blood coagulation indicators in thrombosis group and non-thrombosis group with lung cancer

Index	Thrombosis group (*n*=29)	Non-thrombosis group (*n*=89)	*P*
TM (TU/mL)	10.78 (8.71-13.56)	8.27 (6.81-9.70)	0.000
TAT (ng/mL)	4.15 (1.70-9.66)	1.84 (1.29-2.53)	0.000
PIC (μg/mL)	0.91 (0.68-1.93)	0.76 (0.54-0.95)	0.006
t-PAIC (ng/mL)	7.30 (5.27-12.69)	5.23 (4.16-6.79)	0.007
The data was described as median with interquartile range. TM: thrombomodulin; TAT: thrombin-antithrombin complexes; PIC: *α*2-plasmin inhibitor-plasmin complexes; t-PAIC: tissue plasminogen activator-inhibitor complexes.			

### 2.4 凝血标志物对血栓发生的诊断效能评估

TM、TAT、PIC、t-PAIC、D-D及FDP的曲线下面积分别为0.770、0.771、0.669、0.671、0.819、0.816，差异具有统计学意义（*P* < 0.05）。各项凝血标志物cut-off值、灵敏度、特异度、阴阳性预测值、阴阳性似然比、*Youden*指数见图 1、表 4。

**1:**
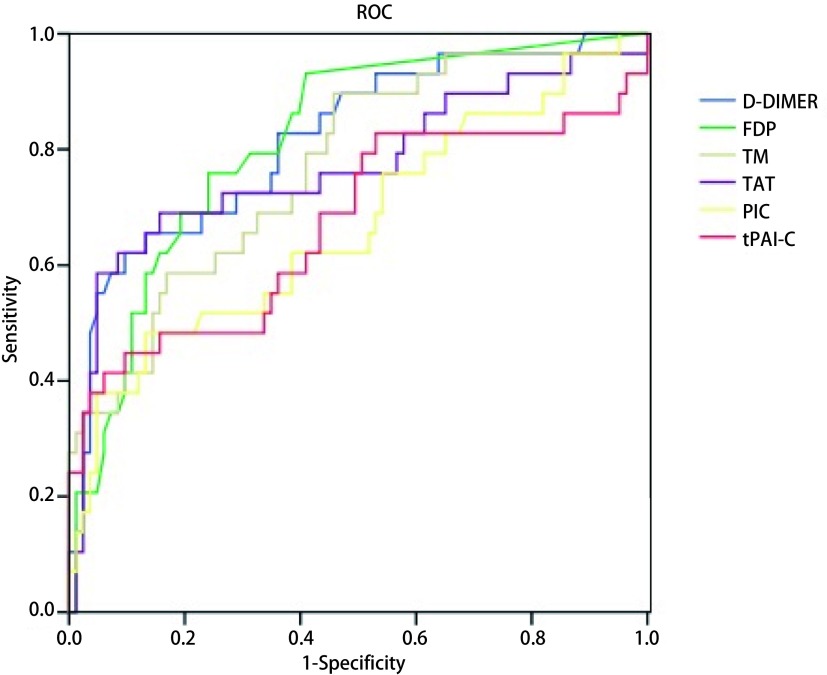
多项凝血纤溶标志物的ROC曲线分析。TM、TAT、PIC、t-PAIC、D-D及FDP的曲线下面积分别为0.770、0.771、0.669、0.671、0.819、0.816（*P* < 0.05） ROC analysis of blood coagulation indicators for diagnosisof thromboembolism. Area under the receiver operating characteristic curve (AUROC) for TM (0.770), TAT (0.771), PIC (0.669), t-PAIC (0.671), D-D (0.819), FDP (0.816) (*P* < 0.05)

**4:** 凝血纤溶标志物的诊断效能评价 Diagnostic performance comparison of blood coagulation indicators

Diagnostic performance	TM	TAT	PIC	t-PAIC	D-D	FDP
The area under the ROC curve (AUC)	0.77^*^	0.771^*^	0.669^*^	0.671^*^	0.819^*^	0.816^*^
Cut-off value	9.40	2.43	0.85	6.30	1.87	5.80
Sensitivity (Sen)	69.0%	72.4%	62.1%	58.6%	72.4%	75.9%
Specificity (Spe)	67.5%	73.5%	61.4%	63.9%	71.1%	75.9%
Youden index	0.37	0.46	0.24	0.23	0.44	0.52
Negative likelihood ratio (NLR)	0.46	0.38	0.62	0.65	0.39	0.32
Positive likelihood ratio (PLR)	2.12	2.73	1.61	1.62	2.51	3.15
Negative predictive value (NPV)	0.86	0.89	0.83	0.83	0.88	0.88
Positive predictive value (PPV)	0.40	0.47	0.35	0.36	0.42	0.43
^*^P < 0.05, compared with AUC of reference line in ROC analysis.						

## 3 讨论

在正常机体中，凝血、抗凝以及纤溶系统的动态平衡是维持体内血液平稳流动状态的重要因素。据报道，血栓栓塞症在普通人群中的发生率仅每年1‰-3‰，但在肿瘤患者中，因疾病本身、手术、放化疗、激素治疗、频繁置管、长期卧床运动减少等多种因素，比率呈4倍-7倍增长，并且取决于不同肿瘤类型和分期^[6]^。

肺癌患者凝血、抗凝与纤溶的平衡在疾病的发生发展过程中也发生偏移，患者体内的高凝状态主要由于肿瘤细胞启动机体炎症反应，生成多种炎性介质及细胞因子直接激活凝血因子^[7]^，同时释放癌性促凝物质，引起血栓的发生^[8]^；肺癌细胞引起血管内皮细胞损伤，高表达的组织因子（tissue factor, TF）释放入血，激活外源性凝血途径，凝血因子激活，凝血酶水平增高；另外，肿瘤患者长期卧床、静脉置管等因素也是血液高凝状态原因。

其次，凝血途径激活，抗凝系统也相应活跃，以稳定机体的凝血功能平衡。本研究中肺癌血栓组TM和TAT水平高于非血栓组，提示在肺癌栓塞症患者体内，TM和TAT水平增高与血栓的发生密切相关。TM是血管内皮损伤的标志物，其水平增高是各种因素对内皮影响的综合反映，肺癌患者内皮损伤后TM释放入血，凝血功能增强，同时机体启动相应代偿机制激活抗凝系统，弥补凝血抗凝纤溶系统平衡的偏移。

最后，随着凝血抗凝途径活跃，纤溶系统亦相应激活。血管内皮细胞释放的凝血酶可引起纤溶系统的被动活化，交联的纤维蛋白水解，继发性纤溶过程形成D二聚体和纤维蛋白原降解产物FDP，侧面反映肺癌患者的血栓形成过程^[9, 10]^。本研究结果发现，肺癌患者血栓组D二聚体和FDP水平高于非血栓组，差异具有统计学意义（*P* < 0.05），也印证了此过程。本研究结果发现，肺癌患者血栓组PIC水平和t-PAIC水平高于非血栓组，差异具有统计学意义（*P* < 0.05），提示在纤维蛋白溶解过程中，患者体内纤溶活性降低，凝血纤溶平衡向血栓形成方向偏移，进一步证实血栓分子标志物的增高与肺癌血栓栓塞症的发生紧密相关^[11]^。

另外，本研究ROC诊断效能分析结果显示，TM、TAT、PIC、t-PAIC、D-D及FDP均对肺癌患者血栓形成有一定诊断价值，其中D-D的曲线下面积（area under curve, AUC）为0.819（*P* < 0.05），优于其余几项凝血标志物，当D-D cut-off值为1.87 mg/L时，灵敏度为72.4%，特异性71.1%。我们认为，肺癌术后置管患者体内多项血栓生物标志物水平升高，反映凝血、抗凝和纤溶活性增强，其变化水平与血栓的发生密切相关。D-D作为继发性纤溶激活的高敏指标，虽灵敏度更高，但本研究的其它五项指标中TM和TAT作为凝血与抗凝系统的代表，ROC曲线下面积均在0.75以上，亦具有较好的诊断效能。提示我们在监测和关注肺癌术后静脉置管患者凝血功能的过程中，更应该综合患者情况，全面关注患者凝血抗凝纤溶，多点监测，以利于预防血栓栓塞症的发生^[12-14]^。

综上所述，肺癌术后置管患者体内凝血和纤溶活性增强，早期监测凝血和纤溶标志物发现患者血液高凝状态，预防抗凝治疗，减少肺癌患者术后血栓并发症的发生，对改善患者预后具有重要意义。
